# Clustering by Fuzzy Neural Gas and Evaluation of Fuzzy Clusters

**DOI:** 10.1155/2013/165248

**Published:** 2013-12-16

**Authors:** Tina Geweniger, Lydia Fischer, Marika Kaden, Mandy Lange, Thomas Villmann

**Affiliations:** Computational Intelligence Group, University of Applied Sciences Mittweida, Technikumplatz 17, 09648 Mittweida, Germany

## Abstract

We consider some modifications of the neural gas algorithm. First, fuzzy assignments as known from fuzzy c-means and neighborhood cooperativeness as known from self-organizing maps and neural gas are combined to obtain a basic Fuzzy Neural Gas. Further, a kernel variant and a simulated annealing approach are derived. Finally, we introduce a fuzzy extension of the ConnIndex to obtain an evaluation measure for clusterings based on fuzzy vector quantization.

## 1. Introduction

Prototype based vector quantization (VQ) is an approved method to cluster and compress very large data sets. *Prototype based* implies that the data are represented by a much smaller number of prototypes. Famous methods are c-means [1], self-organizing maps (SOM) [2], and neural gas (NG) [3]. These methods have in common that each data point is uniquely assigned to its closest prototype. Therefore, they are also called crisp vector quantizers. Yet, in practical applications, data are often overlapping making it hard to separate clusters. For this kind of data fuzzy vector quantizing, algorithms have been developed, for example, fuzzy c-means (FCM) [4] and fuzzy SOM (FSOM) [5]. Now, each datapoint can be partially assigned to each prototype. The FSOM is an extension of the FCM taking the neighborhood cooperativeness into account. Yet, as common to SOM, this neighborhood is bound to an external topological structure like a grid. In this paper we combined FCM with NG, thus exploiting the advantages of each: fuzziness from FCM and dynamic neighborhood cooperativeness without structural restrictions from NG. Our new approach is called Fuzzy Neural Gas (FNG).

Beside its basic functionality we also introduce some variations of FNG. First, we propose the kernel fuzzy neural gas (KFNG) where we consider differentiable kernels to adapt the metric. This allows the algorithm to operate in the same structural space as support vector machines (SVM) [6], which are known to deliver respectable results [7]. In [6], it has been shown that this modified optimization space is equivalent and isometric to a reproducing kernel Hilbert or Banach space, which proves to be beneficial for unsupervised VQ, that is also for FNG.

For another variant of FNG we were inspired by simulated annealing (SA), a method which allows temporary deterioration of an optimization process to stabilize its long term behavior. To obtain an SA-like approach, we introduce *negative learning* and call the new method pulsing Neural Gas (PNG). The idea can also be transferred to FNG resulting in Pulsing Fuzzy Neural Gas (PFNG).

Clustering in general is an ill-posed problem and it is difficult to validate a cluster solution. Specification the validation of very large data sets, where a cluster might be represented by more than one prototype, turns out to be a challenge. There exist a number of validity measures based on separation and compactness, yet most of them presume that each cluster should be represented by exactly one prototype. Ta*ş*demir and Merényi proposed the ConnIndex [8], which is suited to evaluate crisp clusterings, where each cluster contains more than one prototype. This ConnIndex takes the neighborhood structure between the learned prototypes into account to transfer the information of the full data set to the cluster validation process. We propose a modification for fuzzy cluster solutions and use this Fuzzy ConnIndex in the experimental section.

In the experimental section, we use three different data sets, an artificial one and two real world problems, to compare the cluster solutions obtained by FNG with those obtained by FCM. For evaluation purposes the Fuzzy ConnIndex is applied. Further, we demonstrate the performance of Pulsing Neural Gas on a checkerboard data set. This type of problem is highly multimodal and usually the algorithms do not find all clusters.

## 2. Fuzzy Neural Gas

The Fuzzy Neural Gas algorithm is a vector quantizer suitable for overlapping data resulting in fuzzy cluster solutions. It is a combination of the Neural Gas (NG) algorithm which incorporates neighborhood relations between data points and prototypes and the Fuzzy c-Means (FCM) which provides a way to obtain fuzzy data point assignments. In the following section, the NG and the FCM are presented shortly to reproduce the derivation of the FNG originally published in [9]. Besides providing an understanding for the principle functioning of the FNG, the description of the basic algorithms is also useful in preparation of Section 4, where a fuzzy cluster validation method called Fuzzy ConnIndex (fConn) is presented.

### 2.1. Neural Gas

The Neural Gas vector quantizer [3] is an approach which utilizes the dynamic neighborhood between the prototypes *W* = {**w**_*j*_}_*j*=1_^*N*_*P*_^, **w**_*j*_ ∈ ℝ^*d*^, to obtain a clustering of data samples **v**_*i*_ ∈ ℝ^*d*^, *i* = 1,…, *N*_*V*_, from a data set *V*. This neighborhood function is based on a winner ranking of the prototypes for each data point. The rank of prototype **w**_*j*_ is obtained by

(1) $$\begin{array}{c} rk_{j}\left(v_{i},W\right)=\overset{N_{P}}{\underset{l=1}{\sum }}\Theta \left(d\left(v_{i},w_{j}\right)-d\left(v_{i},w_{l}\right)\right), \end{array}$$

with the heaviside function Θ(*x*) = 0, if and only if *x* ≤ 0 and 1 else, and a dissimilarity measure *d*(**v**_*i*_, **w**_*j*_) which determines the distance between data point **v**_*i*_ and prototype **w**_*j*_. Usually the Euclidean distance is used for *d*(**v**_*i*_, **w**_*j*_).

The neighborhood *h*_*σ*_^NG^(**v**_*i*_, **w**_*j*_, *W*) of a data point **v**_*i*_ is specified by

(2) $$\begin{array}{c} h_{\sigma }^{\text{NG}}\left(v_{i},w_{j},W\right)=c_{\sigma }^{\text{NG}}\cdot \exp\left(-\frac{{\left(rk_{j}\left(v_{i},W\right)\right)}^{2}}{2\sigma^{2}}\right), \end{array}$$

where the rank *rk*_*j*_ of prototype **w**_*j*_ is an essential part. For the neighborhood only the prototypes within a certain range *σ* according to their rank are considered, giving the closest prototype the highest emphasis. The constant *c*_*σ*_^NG^ is arbitrarily chosen.

The neighborhood can be used to calculate the local costs:

(3) $$\begin{array}{c} lc^{\text{NG}}\left(v_{i},w_{j},W\right)=h_{\sigma }^{\text{NG}}\left(w_{j},v_{i}\right)\cdot d\left(v_{j},w_{j}\right), \end{array}$$

which resemble the local distortions around prototype *j* weighted by the neighborhood cooperativeness.

The Neural Gas cost function which has to be minimized for optimal clustering directly embeds the local costs:

(4) $$\begin{array}{c} E_{\text{NG}}=\frac{1}{2K\left(\sigma \right)}\overset{N_{P}}{\underset{j=1}{\sum }}\int P\left(v_{i}\right)\underset{lc^{\text{NG}}}{\underset{︸}{h_{\sigma }^{\text{NG}}\left(v_{i},w_{j},W\right)\cdot d(v_{i},w_{j})}}dv_{i}. \end{array}$$

The normalisation constant *K*(*σ*) depends on *c*_*σ*_^NG^ and *P*(**v**_*i*_) is the data density.

The minimization of the cost function (4) is performed by stochastic gradient descent with respect to the prototypes. Given a data point **v**_*i*_ the prototype update rule yields

(5) $$\begin{array}{c} \Delta w_{j}=-\varepsilon \cdot h_{\sigma }\left(v_{i},w_{j},W\right)\cdot \frac{\partial d\left(v_{i},w_{j}\right)}{\partial w_{j}}, \end{array}$$

where *ε* > 0 is the learning rate [3].

After convergence of the algorithm the whole data set *V* is approximated by the set *W* of prototypes. The receptive field *Ω*_*j*_ of each prototype **w**_*j*_ is defined as

(6) $$\begin{array}{c} \Omega_{j}=\left\{v_{i}\;|\;d\left(v_{i},w_{j}\right)<d\left(v_{i},w_{k}\right),\;\;\forall k\right\}. \end{array}$$

For crisp clusterings it has been shown in [3] that the NG algorithm results in better cluster solutions than Self-Organizing Maps (SOM) [2] due to its flexible neighborhood compared to the fixed grid of a SOM.

### 2.2. Fuzzy c-Means

The Fuzzy c-Means [10] is also a vector quantizer where each cluster *Ω*_*j*_ is represented by a prototype **w**_*j*_ located in its center of gravity. Yet contrary to NG, a data point can be assigned to more than one prototype. The cost function to minimize is given by

(7) $$\begin{array}{c} E_{\text{FCM}}=\overset{N_{V}}{\underset{i=1}{\sum }}\;\overset{N_{P}}{\underset{j=1}{\sum }}u_{j}{\left(v_{i}\right)}^{m}d\left(v_{i},w_{j}\right), \end{array}$$

where the fuzzy assignment of data point **v**_*i*_ to prototype **w**_*j*_ is described by *u*_*j*_(**v**_*i*_) ≥ 0. If the restriction ∑_*j*=1_^*N*_*P*_^ *u*_*j*_(**v**_*i*_) = 1 is valid, the clustering is called probabilistic, otherwise possibilistic. The exponent *m* > 1 regulates the fuzziness and according to [10] it should be set to 1.2 ≤ *m* ≤ 2. Again, the distance *d*(**v**_*i*_, **w**_*j*_) is usually chosen to be the Euclidean distance.

The algorithm itself is an alternating optimization of prototypes and fuzzy assignments. The update of the prototypes is carried out by keeping the assignments fixed and vice versa the assignments are adapted based on fixed prototypes:

(8) $$\begin{array}{c} w_{j}=\frac{\sum_{i=1}^{N_{V}}u_{j}{\left(v_{i}\right)}^{m}\cdot \left(\partial d\left(v_{i},w_{j}\right)/\partial w_{j}\right)\;}{\sum_{i=1}^{N_{V}}u_{j}{\left(v_{i}\right)}^{m}}, \end{array}$$

(9) $$\begin{array}{c} u_{j}\left(v_{i}\right)=\frac{1}{\sum_{k=1}^{N_{P}}{\left(d(v_{i},w_{j})/(d(v_{i},w_{k})\right)}^{1/(m-1)}}. \end{array}$$

Since the definition of the receptive field (6) does not reflect the information contained in the fuzzy assignments, we define the fuzzy receptive field as

(10) $$\begin{array}{c} \Omega_{k}^{F}=\left\{v_{i}\;|\;u_{k}\left(v_{i}\right)>0\right\}. \end{array}$$

### 2.3. Combining NG and FCM to the Fuzzy Neural Gas

As mentioned above the Fuzzy Neural Gas can now be obtained by combining NG and FNG. Thereby, the FCM distance function in (7) is replaced by local costs similar to the NG local costs (3):

(11) $$\begin{array}{c} lc_{\sigma }^{\text{FNG}}\left(v_{i},w_{j}\right)=\overset{N_{P}}{\underset{l=1}{\sum }}h_{\sigma }^{\text{FNG}}\left(w_{j},w_{l}\right)\cdot d{\left(v_{i},w_{j}\right)}^{2}, \end{array}$$

yielding the cost function

(12) $$\begin{array}{c} E_{\text{FNG}}=\overset{N_{V}}{\underset{i=1}{\sum }}\;\overset{N_{P}}{\underset{j=1}{\sum }}u_{j}{\left(v_{i}\right)}^{m}\underset{lc_{\sigma }^{\text{FNG}}\left(v_{i},w_{j}\right)}{\underset{︸}{\overset{N_{P}}{\underset{l=1}{\sum }}h_{\sigma }^{\text{FNG}}\left(w_{j},w_{l}\right)\cdot d{\left(v_{i},w_{j}\right)}^{2}}}. \end{array}$$

The local costs (11) take the dynamic neighborhood structure according to

(13) $$\begin{array}{c} h_{\sigma }^{\text{FNG}}\left(w_{j},w_{l}\right)=c_{\sigma }\cdot \exp\left(-\frac{{\left(rk_{j}\left(w_{l},W\right)\right)}^{2}}{2\sigma^{2}}\right) \end{array}$$

into account, where the value *σ* > 0 is the neighborhood range and *c*_*σ*_ assures that ∑_*l*_ *h*_*σ*_^FNG^(**w**_*j*_, **w**_*l*_) = 1. For optimal performance *σ* should be decreased adiabatically in the course of optimization. Note that the neighborhood contrary to the NG neighborhood is based on the winning ranks according to the *best matching prototype* and not as known from NG according to the data. The ranks are calculated similar to (1):

(14) $$\begin{array}{c} rk_{j}\left(w_{l},W\right)=\overset{N_{P}}{\underset{k=1}{\sum }}\Theta \left(d\left(w_{l},w_{j}\right)-d\left(w_{l},w_{k}\right)\right), \end{array}$$

where Θ(*x*) again is the heaviside function.

Analogous to FCM, the update of the prototypes and the fuzzy assignments follows an alternating optimization scheme to minimize the FNG cost function (12). The update scheme consists of two update steps: updating the prototypes while keeping the fuzzy assignments fixed and updating the assignments while retaining the prototypes. The update rules are obtained by Lagrange optimization taking the side condition ∑_*j*=1_^*N*_*P*_^ *u*_*j*_(**v**_*i*_) = 1 into account.

A batch update considering all the data samples at once is possible if the Euclidean distance is used for the calculation of the local costs (11). The resulting equations can be solved for **w**_*j*_ and *u*_*j*_(**v**_*i*_), respectively, yielding

(15) wj=∑i=1NV∑l=1NPul(vi)m·hσFNG(wj,wl)·vi∑i=1NV∑l=1NPul(vi)m·hσFNG(wj,wl),uj(vi) =1∑l=1NP(lcσFNG(vi,wj)/lcσFNG(vi,wl))1/(m−1).

Note that the update of the fuzzy assignments is similar to the FCM assignment update (9) yet instead of the distances *d*(**v**_*i*_, **w**_*j*_) the local costs (11) are considered.

For other distances besides the Euclidean distance, the equation obtained by Lagrange optimization might not be solvable for **w**_*j*_. In that case, the prototypes have to be adapted online via stochastic gradient descent in order to minimize the FNG cost function (12). The corresponding update rule is

(16) $$\begin{array}{c} \Delta w_{j}=\overset{N_{P}}{\underset{l=1}{\sum }}\;\overset{N_{V}}{\underset{i=1}{\sum }}u_{l}{\left(v_{i}\right)}^{m}h_{\sigma }^{\text{FNG}}\left(w_{j},w_{l}\right)\frac{\partial d\left(v_{i},w_{j}\right)}{\partial w_{j}}. \end{array}$$

Since the derivative of the distance ∂*d*(**v**_*i*_, **w**_*j*_)/∂**w**_*j*_ has to be considered, the distance measure is required to be differentiable with respect to **w**_*j*_. Any measure fulfilling this restriction is a suitable measure; that is, alternative to the commonly used Euclidean distance generalized divergences as well as (differentiable) kernels might be used depending on the specific problem at hand. The latter aspect concerning (differentiable) kernels is investigated in detail in the next subsection.

### 2.4. Fuzzy Neural Gas with Differentiable Kernels

For vector quantizers the distance between prototypes and data samples is determined by a distance measure *d*(**v**_*i*_, **w**_*j*_). For FNG this distance has to be differentiable, since the derivative of the distance function ∂*d*(**v**_*i*_, **w**_*j*_)/∂**w**_*j*_ is considered in the prototype update rule (16) to minimize the cost function. This implies that basically any differentiable distance measure is applicable. The common Euclidean distance can be used as well as generalized divergences [11] or (differentiable) kernels [12]. Each reproducing kernel uniquely corresponds to a kernel feature map Φ : *V* → *H*, where *H* is a Hilbert space in a canonical manner [13]. Denote *H*′ = Φ(*V*) to be the image of *V*. The inner product of *H* is consistent with a kernel; that is, *κ*_Φ_(**v**_*i*_, **w**_*j*_) = 〈Φ(**v**_*i*_),Φ(**w**_*j*_)〉_*H*_. Universal continuous kernels ensure the injectivity and continuity of the map. Further, in that case *H*′ is a subspace of *H* [13]. The inner product defines a metric by

(17) dH(Φ(vi),Φ(wj)) =κΦ(vi,vi)−2κΦ(vi,wj)+κΦ(wj,wj).

The nonlinear mapping into the Hilbert space provides large topological richness for the mapped data, which is used for classification in SVMs. However, this topological structure of the image *H*′ may result in better clustering abilities for unsupervised vector quantization.

An example of a universal kernel is the widely known Gaussian kernel:

(18) $$\begin{array}{c} \kappa_{\Phi }\left(v_{i},w_{j}\right)=\exp^{\left(-{||v_{i}-w_{j}||}^{2}/\sigma_{g}^{2}\right)}, \end{array}$$

where ||·|| is the Euclidean norm. This kernel and the distance metric based thereon can be differentiated easily and is therefore suitable to be used with FNG. A disadvantage is that the parameter *σ*_*g*_ has to be estimated, which is known to be a crucial task.

Another simple yet effective kernel is the ELM kernel (extreme learning machine) [14]. The kernel function is defined as

(19) $$\begin{array}{c} \kappa_{\Phi }\left(v_{i},w_{j}\right)=\frac{1}{p}\langle \Phi \left(v_{i}\right),\Phi \left(w_{j}\right)\rangle \end{array}$$

and is simply the normalized dot product in the feature space. In the context of FNG the number *p* of hidden variables corresponds to the number *Z*(*H*′) of intrinsic dimensions [15] of *H*′ with *Z*(*H*′) ≤ *N*. In case that the mapping Φ(*x*) is not known, for *p* → *∞* the kernel can be estimated by an analytic expression [16]:

(20) κΦ(vi,wj)=2πarcsin1+〈vi,wj〉(1/2σ2+1+〈vi,vi〉)(1/2σ2+1+〈wj,wj〉),

which is the so-called asymptotic ELM kernel, where *σ* is the Gaussian distribution of the data.

## 3. Pulsing Neural Gas

It has been shown that the Neural Gas algorithm converges to a global minimum in infinite time [3]. Yet in practice, time is limited and prototypes might only have reached a local minimum by the time the algorithm stops.

The proposed method in this section called Pulsing Neural Gas is a combination of NG and Simulated Annealing (SA), another widely known technique for solving optimization problems. SA is a probabilistic metaheuristics which accepts a random solution with a certain probability following the Boltzmann-Gibbs distribution *p*(Δ, *T*) ~ exp⁡(−Δ/*T*). This probability *p* depends on the difference Δ between a random solution and the former accepted solution and a temperature *T* which is decreasing over time and convergese to zero. Caused by the cooling respective annealing of the temperature *T*, towards the end of the optimization process a deterioration of the cost function is accepted with lower probability than at the beginning. This leads to a stable behavior in the periphery of the global minimum.

To transfer this idea to (Fuzzy) Neural Gas a correspondent to the deterioration in SA has to be found. For the common NG the cost function (4) is minimized by performing stochastic gradient descent learning. Although it cannot be guaranteed, on average the value of the cost function decreases which we consider as *positive learning*. We now introduce *negative learning*; that is, we allow the algorithm to perform a *negative learning step*, which increases the cost function temporarily. Hence, on average, the algorithm performs *positive learning*, but once in a while with a certain decreasing probability following a Gibbs-distribution a *negative learning step* causes a disturbance. Possibly this helps to overcome local minima and speeds up convergence to the global minimum.

**Figure 1:**
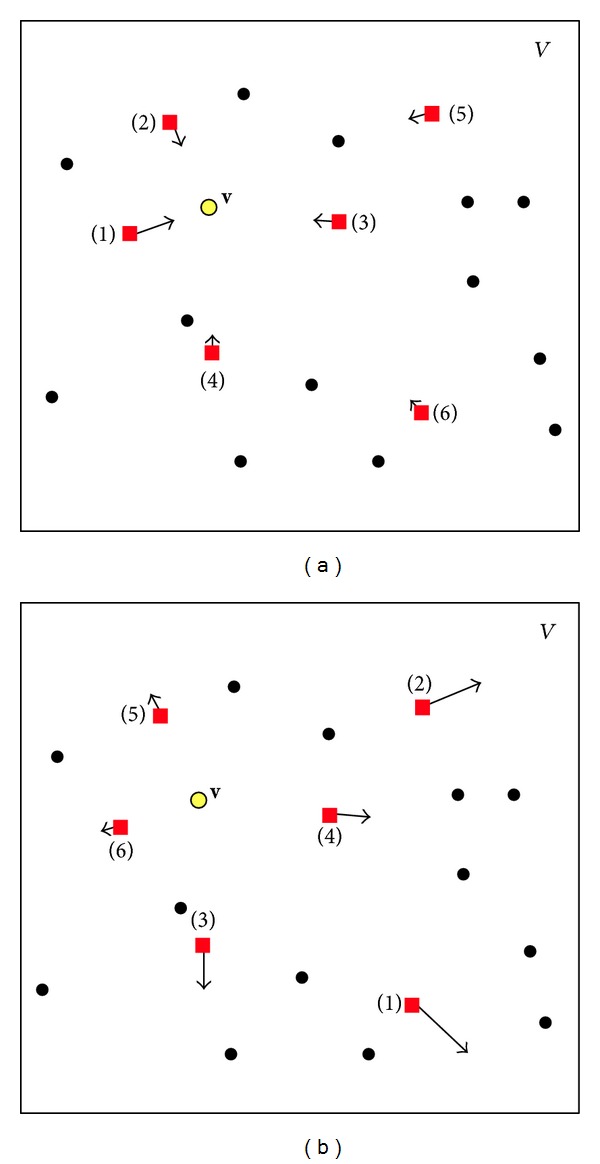
Prototype update Neural Gas (a) versus Pulsing Neural Gas (b). NG: the closer the prototype the lower its rank and the stronger the update in direction of the data point. PNG: at random time steps *negative learning* is performed. The closer the prototype the higher its rank and the weaker the update in the opposite direction of the data sample.

First considerations took gradient ascent learning into account. However, investigations have shown that this strategy leads to an unstable learning behavior. Instead we suggest a *reverse prototype ranking*:

(21) $$\begin{array}{c} rk_{j}^{-}\left(v_{i},W\right)=\left(N_{p}-1\right)-\overset{N_{p}}{\underset{k=1}{\sum }}H\left(d\left(v_{i},w_{j}\right)-d\left(v_{i},w_{k}\right)\right), \end{array}$$

for a given data point **v**_*i*_. This ranking reverses the known (positive) ranking (1) such that the prototype with the largest distance now becomes the best (lowest) rank (see Figure 1); that is, the update of the prototypes is performed in reverse order and in opposite direction. The prototype update rule is formulated as

(22) $$\begin{array}{c} \Delta w_{j}=\varepsilon \cdot h_{\sigma }^{-}\cdot \frac{\partial d\left(v_{i},w_{j}\right)}{\partial w_{j}}, \end{array}$$

where the neighborhood function

(23) $$\begin{array}{c} h_{\sigma }^{-}\left(j,l\right)=c_{\sigma }\cdot \exp\left(-\frac{{\left(rk_{j}^{-}\left(w_{l},W\right)\right)}^{2}}{2\sigma^{2}}\right) \end{array}$$

depends on the reverse rankings *rk*_*j*_^−^ (21). Now, in contrast to the common positive NG update step, the prototypes are not moved towards the presented data point. Yet instead, according to their reverse ranks they are pushed away, causing little change on the prototypes close to the data point and larger shifts of the prototypes located farther away. Figure 1 depicts this difference between the common NG and the Pulsing NG incorporating negative learning motivated by Simulated Annealing.

Unfortunately, this strategy is not directly transferable to the batch variants of NG [17] and FNG (12). Here all the data points are presented at once and the relocation of the prototypes at each update step depends on all data points. For this variant the idea of Simulated Annealing is performed differently. Instead of a reverse ranking, now only a random subset of the data samples is presented at a randomly chosen update step:

(24) $$\begin{array}{c} w_{j}=\frac{\sum_{v\in A}h_{\lambda }\left(rk_{j}\left(v,W\right)\right)\cdot v}{\sum_{v\in A}h_{\lambda }\left(rk_{j}\left(v,W\right)\right)}, \end{array}$$

where *A* ⊂ *V* is a nonempty subset. The probability for performing this update step again follows a Gibbs-distribution decreasing with proceeding training. This way, the trend of the relocations is interrupted enabling the prototypes to leave prospective local minima yet possibly causing higher costs temporarily.

One can visualize this procedure as a more or less smooth process approximating some local optimum and once in a while the whole system is shaken up resulting in a temporary increase of the cost function and causing a reorientation of the whole adaptation process. We name this modification of the NG algorithm Pulsing Neural Gas (PNG) and for the fuzzy variant FPNG.

## 4. Fuzzy ConnIndex for the Evaluation of Fuzzy Clusterings

A strategy to cluster very large data sets is to perform vector quantization followed by a clustering of the obtained prototypes. If it can be assured that each of the resulting clusters *Ξ*_*l*_ is represented by more than one prototype, the ConnIndex [8] as proposed by Ta*ş*demir and Merényi can be used for validation purposes. Yet, the ConnIndex is suitable only if crisp vector quantization has been performed in the first step. Since we need a method to evaluate cluster solutions based on fuzzy vector quantization we modified the original ConnIndex. In the following, first we recapitulate the index as proposed by Ta*ş*demir and Merényi and subsequently we derive a fuzzy version of the ConnIndex.

*Original ConnIndex*. In general, the original ConnIndex balances the overall cluster compactness and separation by combining the intercluster connectivity *C*_inter_ ∈ [0,1] and the intracluster connectivity *C*_intra_ ∈ [0,1]:

(25) $$\begin{array}{c} C=C_{intra}\cdot \left(1-C_{inter}\right). \end{array}$$

Thereby, *C*_*intra*_ measures the compactness of the clusters and *C*_*inter*_ evaluates the separation between them. A value of *C* close to one suggests a good cluster solution.

For the estimation of the connectivity a nonsymmetric cumulative adjacency matrix **A**

(26) $$\begin{array}{c} A=\overset{N_{V}}{\underset{i=1}{\sum }}\Psi \left(v_{i}\right) \end{array}$$

with respect to the receptive fields *Ω*_*j*_ (6) is considered. Here, Ψ(**v**_*i*_) is the zero (*N*_*P*_ × *N*_*P*_)-matrix except the element Ψ_*s*_0_,*s*_1__ which refers to the best matching unit *s*_0_(**v**_*i*_) = argmin⁡_*j*_ (*d*(**v**_*i*_, **w**_*j*_)) and the second best matching unit *s*_1_(**v**_*i*_) = argmin⁡_*k*∣*k*≠*s*_0__(*d*(**v**_*i*_, **w**_*k*_)) for data point **v**_*i*_. The value of this element is set to a positive constant *γ*_1_ usually chosen as *γ*_1_ = 1. The matrix Ψ(**v**_*i*_) is called the response matrix with respect to the data vector **v**_*i*_. As pointed out in [8], the row vector **a**_*j*_ = (*a*_*j*,1_,…, *a*_*j*,*N*_*P*__) of **A** describes the density distribution within the receptive field *Ω*_*j*_ with respect to the other *N*_*P*_ − 1 prototypes.

The symmetric connectivity matrix

(27) $$\begin{array}{c} C=A+A^{Τ} \end{array}$$

reflects the topological relations between the prototypes based on the receptive field evaluation. Thereby, the elements *c*_*j*,*k*_ reflect the dissimilarities between the prototypes based on the local data densities.

Now, having the matrices **A** and **C** defined, the before mentioned connectivities *C*_*intra*_ and *C*_*inter*_ can be evaluated. The intracluster connectivity *C*_*intra*_ is based on the cumulative adjacency matrix **A** (26):

(28) $$\begin{array}{c} C_{intra}\left(l\right)=\frac{\sum_{j,k\;|\;j\neq k}\left\{a_{j,k}\;|\;w_{j},w_{k}\in \Xi_{l}\right\}}{\sum_{j,k\;|\;j\neq k}\left\{a_{j,k}\;|\;w_{j}\in \Xi_{l}\right\}} \end{array}$$

for each cluster *Ξ*_*l*_. The greater the compactness of a cluster *Ξ*_*l*_ the closer its intraconnectivity is to one. Note again that, as mentioned above, each cluster is made up of more than one prototype **w**_*j*_.

The inter-cluster connectivity *C*_*inter*_ evaluates the separation between the clusters. Analogously, it is the average over the local inter-cluster connectivities

(29) $$\begin{array}{c} C_{inter}\left(l\right)=\underset{1\leq m\leq K,m\neq l}{\max}C_{inter}\left(l,m\right) \end{array}$$

of all clusters evaluating the separation of each cluster *Ξ*_*l*_ to the other clusters *Ξ*_*m*_, *m* ≠ *l*. Thereby, *C*_*inter*_(*l*, *m*) judges the separation of cluster *Ξ*_*l*_ to cluster *Ξ*_*m*_ based on the connectivity matrix **C** (27) and is defined as

(30) Cinter(l,m) ={0if  Sl,m=0 ∑j,k ∣ j≠k{cj,k ∣ wj∈Ξl,wk∈Ξm}∑j,k ∣ j≠k{cj,k ∣ wj∈Sl,m}if  Sl,m≠0,

where the sets

(31) $$\begin{array}{c} S_{l,m}=\left\{w_{j}\;|\;w_{j}\in \Xi_{l}\wedge \exists w_{k}\in \Xi_{m}:a_{j,k}>0\right\} \end{array}$$

describe the neighborhood relations between the clusters *Ξ*_*l*_ and *Ξ*_*m*_ based on the contained prototypes. In contrast to *C*_*intra*_, the value of *C*_*inter*_ decreases with better separability.

*Generalization of the ConnIndex*. The ConnIndex by Ta*ş*demir and Merényi considers the best and second best matching units *s*_0_(**v**_*i*_) and *s*_1_(**v**_*i*_) only, discarding any information provided by higher ranked prototypes. A generalized version of the index is obtained by incorporating higher winning ranks as known from Neural Gas [3]; see (1). Obviously *rk*_*s*_0_(**v**_*i*_)_(**v**_*i*_, *W*) = 0 is the rank of the best matching prototype. Analogously, the *p*th winner is denoted by *s*_*p*−1_(**v**_*i*_) with rank *rk*_*s*_*p*__(**v**_*i*_, *W*) = *p*. If it is clear from the context, we will abbreviate *s*_*p*_ = *s*_*p*_(**v**_*i*_) in the following.

To incorporate the higher winning ranks the response matrix Ψ(**v**_*i*_) has to be redefined to involve the *full* response of the whole vector quantizer model for a given input **v**_*i*_. The new response matrix $\bar{\Psi }(v_{i})$ is a zero matrix of the same size as Ψ(**v**_*i*_) except the row vector regarding the winner *s*_0_(**v**_*i*_). The new response matrix is set to

(32) $$\begin{array}{c} {\bar{\Psi }}_{s_{0}}\left(v_{i}\right)=r\left(v_{i}\right), \end{array}$$

where **r**(**v**_*i*_) is the so-called response vector of all prototype responses for a given input **v**_*i*_. The vector elements *r*_*j*_(**v**_*i*_) of the *j*th prototype are defined as

(33) $$\begin{array}{c} r_{j}\left(v_{i}\right)=\varphi_{\omega }\left(rk_{j}\left(v_{i},W\right)\right), \end{array}$$

with *φ*_*ω*_(*x*) being an arbitrary monotonically decreasing function in *x*. A simple choice for this function is the exponential function *φ*_*ω*_(*x*) = exp⁡(−(1 − (*id*(*x*))/(*n* − 1))/2*ω* ^2^). The parameter *ω* determines the range of influence and should be determined carefully. If for the vector quantization an algorithm incorporating neighborhood cooperativeness in learning like Neural Gas [3] or self-organizing maps [18] was used, the *ω*-parameter should be chosen according to the neighborhood range used there. Yet, an alternative approach could be the direct utilization of the distances *d*(**v**_*i*_, **w**_*j*_) instead of the winning ranks and *φ*_*ω*_(*x*).

This generalized version of the ConnIndex *C*_*g*_ uses for the calculation of the cumulative adjacency matrix **A** in (26) the new response matrices $\bar{\Psi }(v_{i})$ instead of the original response matrices Ψ(**v**_*i*_).

Note that this version is in concordance with the original version, if *φ*(*x*) is chosen as

(34) $$\begin{array}{c} \varphi \left(x\right)=\{\begin{array}{cc} \gamma_{0} & \text{for}\;x=0\\ \gamma_{1} & \text{for}\;x=1\\ 0 & \text{else}. \end{array} \end{array}$$

*Fuzzy ConnIndex*. Up to now we assumed that the vector quantization model is based on a crisp mapping. For these models a winner ranking is available and the response information of the network is collected in the response vector **r**(**v**_*i*_), reflecting the topological relation between the prototypes. In fuzzy vector quantization algorithms this information is no longer available because each data point is gradually assigned to all prototypes. Yet, the fuzzy data point assignments *u*_*j*_(**v**_*i*_) which can be stored in a *N*_*V*_ × *N*_*P*_ assignment matrix *U* also reflect the topography of the underlying data. The assignment vector **u**_*i*_ is then the specific vector of *U* which contains the assignment value of a data point **v**_*i*_ to all of the prototypes and is comparable to the response vector **r**(**v**_*i*_) used for the Generalized ConnIndex *C*_*g*_. Therefore, the assignments can be used directly to determine the response matrix $\bar{\Psi }(v_{i})$ by substituting the response vector ${\bar{\Psi }}_{s_{0}}(v_{i})$ in (32). Consequently, the best matching prototype *s*_0_(**v**_*i*_) for a given data vector can be seen as the prototype with the highest fuzzy assignment *u*_*j*_(**v**_*i*_):

(35) $$\begin{array}{c} s_{0}=\underset{j}{\max}\left\{u_{j}\left(v_{i}\right)\right\}. \end{array}$$

Now, the row vector ${\bar{\Psi }}_{s_{0}}(v_{i})$ of the redefined response matrix $\bar{\Psi }(v_{i})$ can simply be chosen as the fuzzy response vector **u**_*i*_:

(36) $$\begin{array}{c} {\bar{\Psi }}_{s_{0}}\left(v_{i}\right)=u_{i}. \end{array}$$

Again, the cumulative adjacency matrix **A** is calculated as before for the original ConnIndex *C* and the Generalized ConnIndex *C*_*g*_ according to (25). Further calculations remain unaffected.

Hence, the resulting new fuzzy ConnIndex *C*_*f*_ is the counterpart of the generalized ConnIndex *C*_*g*_ in case of fuzzy vector quantization models.

## 5. Performance

To evaluate the performance of FNG we designed different experiments to compare this method with crisp vector quantizers and the fuzzy c-Means. We also conducted an experiment examining the pulsing FNG. To perform the tests we used artificial and real world data sets.

For the evaluation of the cluster results we used the ConnIndex or the Fuzzy ConnIndex, respectively. This evaluation measure, described in the previous section, is relatively new [8, 19]. But it seems to be well suited for the evaluation of cluster solutions in terms of separation and compactness.

Additionally, for the first *Smiley* dataset we also calculated the Kappa value *κ*_*C*_ [20] which is a measure to judge the agreement of two cluster solutions. A variant thereof *κ*_*F*_ is suitable for fuzzy data [21]. Unfortunately, this measure can be used only for cluster solutions with a low number of clusters, since the clusters of the different solutions have to be matched, which is hard for clusterings containing a higher number of clusters.

### 5.1. Artificial Dataset:*Smiley*

**Figure 2:**
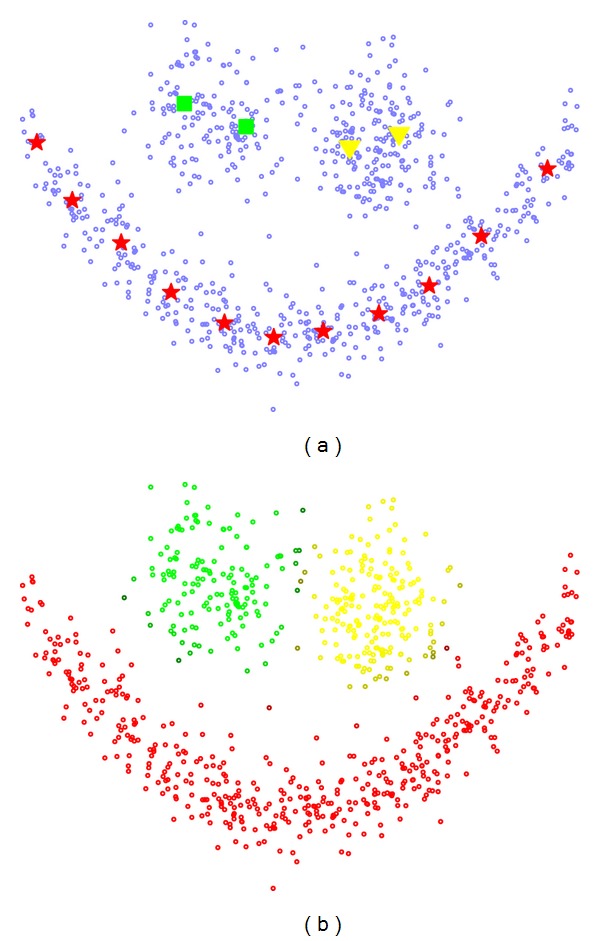
*Smiley* dataset clustered by Fuzzy Neural Gas (FNG). (a) Obtained prototypes. (b) Fuzzy cluster assignments of the data points.

In the first setting we used the *Smiley* data set [19]. This two-dimensional data set consists of three clusters with varying shapes, number of data samples, variances, and distances to each other (see Figure 2). It contains a total of 809 data points.

In the first step we apply c-Means and NG to perform crisp vector quantization and FCM and FNG to perform fuzzy vector quantization with the fuzziness parameter *m* set to different values *m* = {1.1,…, 2.0}. All algorithms result in acceptable solutions. For *m* = 1.4 the FNG cost function settles at the lowest value; FCM reaches the lowest costs for *m* = 1.5. The obtained FNG prototypes are depicted in Figure 2; the FCM results look similar. Visual evaluation confirms an intuitively good distribution in the data space.

A more objective evaluation is obtained with the help of the (Fuzzy) ConnIndex. Yet, to apply this measure the prototypes themselves have to be grouped to clusters of at least two prototypes each. In this simple experiment this step is done manually following the inherent obvious structure of the data set consisting of three clusters.

The obtained ConnValues are listed in Table 1 and show as expected a clear discrepancy between the ConnIndex values obtained by crisp and those obtained by fuzzy vector quantization. This is due to the influence of the data points located in the gaps between the main clusters on the calculation of the index. It is also evident that NG and FNG perform better than c-Means and Fuzzy c-Means, respectively. The overall best ConnIndex value is obtained for FNG, which is less surprising since this algorithm is a combination of FCM and NG, taking beneficial features of each: NG neighborhood and FCM fuzzy assignments.

The Kappa values *κ*_*C*_ [20] and *κ*_*F*_ [21] measure the agreement of two cluster solutions. The closer the values are to one, the higher is the agreement. Comparing the given data structure with the results obtained by the four different clustering methods yields high values indicating substantial to perfect agreement (according to [22]); see Table 1. It can be observed that the two crisp methods NG and c-Means performed almost equally, while the discrepancy between FCM and FNG is remarkable, indicating superior performance of FNG. Note that the values of the crisp and fuzzy solutions cannot be compared to each other since two different *κ*-measures are applied.

### 5.2. Practical Example: *Indian Pine*

*Indian Pine* is a publicly available data set taken by the NASA Airborne Visible/Infrared Imaging Spectrometer (AVIRIS) consisting of 145 × 145 pixels [23]. Data samples cover 220 bands of 10 nm width from 400 to 2500 nm. Due to the atmospheric water absorption 20 noisy bands can be identified (104–108, 150–163, and 220) and removed safely [24]. The data set is labeled according to 16 identified classes, but we do not use this information for the current experimental setting.

The processing of the data consists of two steps. First vector quantization is performed to position the prototypes. For this step the same algorithms as in the last experiment are used: crisp c-Means and NG and fuzzy FCM and FNG. In the second step the obtained prototypes are grouped by affinity propagation (AP) [25] to be able to apply the (Fuzzy) ConnIndex for evaluation. Special care has to be taken to fulfill the requirement that each cluster (i.e., prototype cluster) is represented by more than one prototype. For this reason a sufficiently high number of prototypes has to be chosen. We set this number to 64 (four times the number of known classes).

The calculation of the Generalized ConnIndex *C*_*G*_ for the crisp methods is straightforward. For the fuzzy variants again the fuzziness parameter *m* has to be considered carefully. A value of *m* = 1.5 has proven to be favourable. The respective obtained ConnIndex values *C*_*G*_ and *C*_*F*_ are listed in Table 1.

Although the prototype clustering by Affinity Propagation always results in crisp cluster assignments, the clustering based on FNG vector quantization still yields better ConnIndex values than the other methods.

### 5.3. Practical Example: *Colorado*

The *Colorado* data set [26] is a LANDSAT TM image from the Colorado area, USA. The image covers a region of about 50 × 50 kilometers yielding approximately 2 million data points. These are labeled by experts according to 14 different vegetation types and geological formations found in this region. Among them are aspen, mixed pine forest, water, moist meadow, and dry meadow to name a few. The original data samples are 7-dimensional, yet one band (thermal band) is removed due to its low resolution. Generally, the bands are highly correlated [26].

For the experiment we neglected the class information and selected randomly 10% of the data with a representative class distribution. The number of prototypes is set to 56. Besides that, the setup of the experiment is identical to the setup for the *Indian Pine* and consists of the two there described processing steps.

It can be observed that the FCM training is much faster than FNG training and requires less training cycles, about 35 versus 90. Yet, the Fuzzy ConnIndex yields much better results for FNG than FCM (see Table 1) indicating a better prototype distribution in terms of inter- and intraconnectivity of the obtained clusters. The reason for the prolonged processing time can be found in the computational costs to calculate all neighborhood relations anew in each processing step.

### 5.4. Artificial Data Set: *Checkerboard*

**Figure 3:**
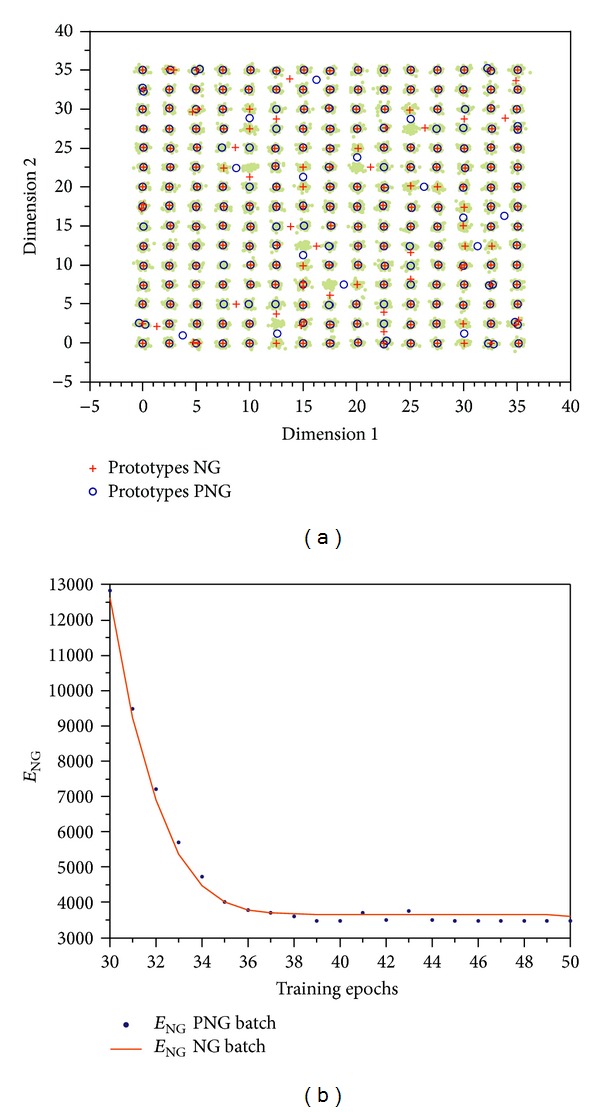
Final cluster solutions (a) and cost functions (b) for the *Checkerboard* data set using NG and PNG. (a) It can easily be verified that the PNG algorithm has more prototypes placed within the clusters than common NG. The number of training steps for both algorithms was the same. (b) The blue dots refer to the costs for Pulsing Neural Gas. The little bumps at time steps 41 and 43 indicate that a *negative learning* step has occurred.

This artificial data set [27] consisting of compact yet well-separated clusters arranged in a checkerboard-like manner is well suited to demonstrate the performance of the Pulsing Neural Gas compared to the common Neural Gas. The data set contains 11.250 two-dimensional data vectors, which are grouped in 15 × 15 normally distributed clusters with a standard deviation of *σ* = 0.3. The mean distance between two neighboring cluster centers is 2.5. Due to the low dimensionality the data set is well suited for visualization; see Figure 3(a).

For both algorithms NG and PNG all 225 prototypes are initialized in the center of the data set. In the following the algorithms are run both for the same number of steps. For comparison the values of the energy functions according to (4) are used. The experiment showed that on the long run both algorithms performed well. For online learning the effect of the pulsing variant is neglectable, yet the batch version shows significant improvements. The cost function of the Pulsing Neural Gas reaches lower values. The negative learning steps show as little bumps in the plot of the energy functions (see Figure 3(b)), indicating a temporary deterioration. In Figure 3(a) the prototype distribution after 50 learning steps is visualized. Obviously the number of misplaced NG prototypes is higher than the number of misplaced PNG prototypes. This finding is in accordance with the lower value of the PNG energy function.

## 6. Conclusion

We proposed in this paper a fuzzy version of the Neural Gas. By combining the concept of neighborhood cooperativeness as known from NG with the FCM fuzzy assignments we obtain the Fuzzy Neural Gas. This algorithm outperforms FCM by taking dynamic neighborhood relations into account, a paradigm proven to be well suited for crisp vector quantization. The resulting FNG shows good performance compared to standard FCM and crisp NG. Due to the neighborhood cooperativeness this algorithm is insensitive to the initialization of the prototypes.

It is straight forward to introduce other distance measures besides the commonly used Euclidean distance. The only prerequisite is that the measure has to be differentiable; for example, differentiable kernels might be used.

A further variant of NG, respectively, its fuzzy version, is the Pulsing Neural Gas imitating a Simulated Annealing-like behaviour. This modification which allows temporary deterioration of the cost function stabilizes in the long run the learning procedure and helps the algorithm to overcome local minima more easily. This effect was demonstrated on a checkerboard data set, for which it is known that usually the algorithms do not find all clusters.

And finally, we extended the original crisp cluster evaluation ConnIndex [21] to be used for fuzzy clustering. It is based on a generalization of the index considering all prototypes instead of first and second best matching units only. The fuzzy version additionally takes the fuzzy information provided by the fuzzy data point assignments into account. As the original, the Fuzzy ConnIndex requires more than one prototype per cluster. The index was used for the evaluation of the experiments.

## Figures and Tables

**Table 1:** ConnIndex and *κ*-values for clusterings of different datasets obtained by crisp and fuzzy vector quantizers. Due to the low number of clusters of the *Smiley* dataset the *κ*-values can be calculated. There is almost no difference between the crisp methods but a substantial discrepancy between the fuzzy vector quantizer. FNG performs way better than FCM. This observation is supported by evaluating the ConnIndex. To evaluate the cluster solutions of the other two datasets only the ConnIndex was used: for crisp methods the generalized ConnIndex *C*_*G*_ and for fuzzy methods the Fuzzy ConnIndex *C*_*F*_.

	*Smiley*		Indian Pine ConnIndex	Colorado ConnIndex
	*κ*-value	ConnIndex		
Crisp				
CM	*κ*_*C*_ = 0.978	*C*_*G*_ = 0.1478	*C*_*G*_ = 0.13	*C*_*G*_ = 0.2321
NG	*κ*_*C*_ = 0.980	*C*_*G*_ = 0.2634	*C*_*G*_ = 0.21	*C*_*G*_ = 0.4207
Fuzzy				
FCM	*κ*_*F*_ = 0.775	*C*_*F*_ = 0.6279	*C*_*F*_ = 0.65	*C*_*F*_ = 0.6042
FNG	*κ*_*F*_ = 0.953	*C*_*F*_ = 0.9272	*C*_*F*_ = 0.72	*C*_*F*_ = 0.7228
